# Mitochondrial dynamism and the pathogenesis of Amyotrophic Lateral Sclerosis

**DOI:** 10.3389/fncel.2015.00031

**Published:** 2015-02-10

**Authors:** Mauro Cozzolino, Simona Rossi, Alessia Mirra, Maria Teresa Carrì

**Affiliations:** ^1^Institute of Translational Pharmacology, CNRRome, Italy; ^2^Department of Biology, Università di Roma Tor VergataRome, Italy; ^3^Fondazione Santa Lucia IRCCSRome, Italy

**Keywords:** ALS, mitochondria, FTD, C9orf72, CHCHD10

## Abstract

Research on mitochondria in the last years has been characterized by the fundamental finding that the morphology of mitochondria is deeply connected to the regulation of a vast number of different processes, including oxidative phosphorylation and ATP production, calcium buffering, and apoptosis. This has immediately focused the attention of the neuroscience community to the possible involvement of mitochondrial dynamism, the process underlying morphological features of mitochondria, in neurodegeneration, where mitochondrial dysfunction is believed to represent an important contributing event, or even a primary causative factor. Amyotrophic Lateral Sclerosis (ALS), a disease of motor neurons and their neighboring cells, has long been considered as a neurodegenerative disease with an important mitochondrial issue. Yet, whether mitochondria have a causative, primary role in the pathogenic process has always been debated, and the specific defects which account for this role are elusive. Here we discuss recent genetic advances suggesting that defective mitochondrial dynamism is primarily involved in the pathogenic mechanisms of ALS, and that foster the longstanding concept that disruption of mitochondrial function is a vulnerable factor for motor neurons.

## Introduction

Demise of motor neurons in the spinal cord, brain cortex, and brainstem of patients is responsible for Amyotrophic Lateral Sclerosis (ALS). Degeneration of motor neurons has been considered for a long time the most significant feature of ALS. However, since the discovery of the first gene responsible for familial forms of ALS, *Sod1*, many fundamental advances in our knowledge of the disease have been reached, and the comprehension of the biological, genetic and clinical processes underlying ALS, has impressively increased (Cozzolino et al., 2012). According to these findings, the current thinking is that ALS is a complex multi-systemic and multi-factorial syndrome and that motor neuron degeneration is only part of a more widespread and multifaceted disease process. Different lines of evidence support this concept. First, ALS appears as a multi-systemic disease, where different tissues and cell types, such as astrocytes and microglia, oligodendrocytes and muscles cells, among others, are both targeted by the disease processes and actively participate to the final disease outcome (Robberecht and Philips, 2013). Second, a number of genes have been identified so far that are definitely associated to ALS pathogenesis and that now cover a large fraction of the familial cases of the disease (Renton et al., 2014). According to their known biological role, or to new acquired functions as a consequence of gene mutations, they are apparently connected to different physiological processes, implying that diverse pathogenic mechanisms, not necessarily related, might be involved in ALS onset and progression. Indeed, robust experimental evidence showing that RNA dys-regulation, protein misfolding, oxidative damage, defective axonal transport and excitotoxicity are actively implicated in ALS has accumulated from studies in experimental models as well as tissue samples and iPSC-derived neural cells from patients (Cozzolino et al., 2012). Third, many of the same gene mutations that are definitely involved in familial forms of ALS (and even in apparently sporadic forms, which represent the vast majority of ALS cases) have been also described as associated to diseases that do not primarily manifest with motor neuronal phenotypes, and in particular to Fronto Temporal Dementia (FTD; Cooper-Knock et al., 2014), strengthening the complex character of the disease and the consequent variability in its phenotypic expression, which is now well recognized (Swinnen and Robberecht, 2014).

In the very last years, the major focus of ALS research has moved to RNA control of motor neuron functions, as most of the newly identified genes are more or less directly associated to RNA regulation. These include FUS/TLS and TDP-43, two DNA/RNA binding proteins with an established, yet not completely clear, role in the regulation of RNA transcription, splicing, transport and translation, and *C9orf72*, a gene that is profoundly affected by the presence, in carriers, of an highly expanded GGGGCC repeat: it is widely believed that this repeat provides the mutant gene of an acquired, toxic feature by an RNA-dependent gain of function mechanism (Achsel et al., 2013). Since these three genes alone account for more than half of ALS familial cases, RNA dys-metabolism is likely to represent a central issue in ALS pathogenesis. Yet, as we will discuss in the next paragraphs, recent genetic evidence put mitochondria, an old player in ALS research, under the spotlight once again.

## Mitochondria involvement in ALS: functional evidence and traits of an (un)resolved issue

Mitochondrial dysfunction has always been recognized as a candidate major player in ALS pathogenesis. Abnormal mitochondrial structure, alterations in the electron transport by mitochondrial complexes I-IV, defects in the import of mitochondrial proteins, to cite just some of the key features of mitochondria, have been found in ALS models and patients (for comprehensive reviews, see Cozzolino and Carrì, 2012; Tan et al., 2014). As a result, cellular energy imbalance, increased oxidative stress, induction of apoptosis, and impaired calcium handling (all traits that have been frequently observed in cell, animal models, and, as far as it is possible, also in patients) have been considered as likely direct consequences of mitochondrial dys-functioning. Most notably, many of these mitochondrial defects can be clearly detected much before the onset of symptoms, arguing for a primary role of mitochondria in the disease determination. Even some of the proteins that are definitely responsible for, or at least associated to familial ALS cases, have been described to possess mitochondria-related function. This is the case of SOD1, a protein with a prevalent steady state cytosolic localization, and with a small fraction localized in the intermembrane space of mitochondria, where it possibly exerts an antioxidant function; VAPB, an endoplasmic reticulum (ER)-resident protein that regulate ER-mitochondria interaction; and optineurin, that is recruited to damaged mitochondria that are then targeted to the mitophagic process underlying mitochondria recycling. ALS associated mutations are believed to either disrupt these functions, as in the case of VAPB and optineurin, or to confer new functions that are detrimental to mitochondria, as in the case of SOD1 and TDP43 (Cozzolino et al., 2013; Stoica et al., 2014; Wong and Holzbaur, 2014).

Yet, clinical testing of mitochondrial protecting drugs was in no instance able to provide some beneficial effect to patients, although positive outcomes could be predicted by the effects that the same drugs exerted on ALS models (Mitsumoto et al., 2014; Poppe et al., 2014). Even preclinical genetic approaches aimed at supporting a causal role for mitochondrial function disturbance in ALS gave conflicting results. For example, preserving spinal cord mitochondrial function by removal of p66Shc, a key regulator of mitochondrial oxidative balance, provided a very significant delay in both disease onset and life span of G93A-SOD1 mice (Pesaresi et al., 2011). However, when mitochondrial function was robustly restored by increasing the calcium-buffering activity of mitochondria through cyclophylin D knock-out, no significant effect on muscle denervation, motor axon degeneration, disease progression and survival was observed, questioning whether mitochondria do indeed participate to the disease (Parone et al., 2013).

In view of the key role of morphology regulation in determining the functional state of the mitochondria, and considering that dysfunctions in this process are clearly associated to neurodegeneration (Chen and Chan, 2009), including motor neuron degeneration (Züchner et al., 2004), it is not surprising that a number of efforts have been made to ascertain whether mitochondrial dynamism defects could be involved in ALS disease. This has been essentially performed in cell and animal models based on mutant SOD1s expression, but other disease models that express newly identified ALS genes have been tested as they became available.

Fusion and fission are responsible for the overall structure of mitochondria. The two processes are highly coordinated and strictly controlled by a series of dedicated proteins, whose number is continuously increasing. The balance between the activity of fusion and fission proteins thus determines the shaping of the mitochondrial network, which in turn has a central role in mitochondrial function in all tissues, including the nervous tissue (Itoh et al., 2013; da Silva et al., 2014). A clear fragmented mitochondrial network is induced by the expression of different SOD1 mutants in a variety of cellular models, including rat spinal cord motor neurons (Magrané et al., 2009; Ferri et al., 2010; Pesaresi et al., 2011; Song et al., 2013). Accordingly, the expression of the pro-fusion proteins Mfn1 and Opa1, and the pro-fission proteins Drp-1 and Fis-1, is respectively decreased, or maintained at high levels during the disease course that characterizes mutant SOD1 mice (Liu et al., 2013). Importantly, preservation of mitochondrial function by overexpression of PGC-1alpha, Sirtuin-3 or Glutaredoxin-2, three proteins that are known to regulate intracellular ROS balance and mitochondrial energy metabolism, restores a correct mitochondrial network, as well as the steady state expression levels of mitochondrial dynamism proteins (Ferri et al., 2010; Song et al., 2013). Therefore, also in ALS an imbalance between fusion/fission regulation might well be involved in mitochondrial dysfunction. Most notably, overexpression of a dominant negative form of DRP1 which lacks the GTPase activity and thus blocks mitochondrial fission, is able to inhibit mitochondrial fragmentation and cell death in cortical neurons expressing mutant SOD1, indicating that, at least in this experimental system, a causal link exists between defects in the fusion/fission process and the functional and structural mitochondrial alterations (Song et al., 2013).

A bulk of experimental evidence has indicated important functional links between the dynamism of mitochondria and their transport along axons, and this concept also emerged from studies where mitochondrial morphology alterations and mitochondrial transport have been analyzed in the context of ALS-related cultured cells and mouse models. Impairment of axonal transport of mitochondria is an early event in mice overexpressing ALS-linked mutant forms of SOD1 or wild-type and mutant TDP43, all mimicking, to different degrees, the disease (De Vos et al., 2007; Wang et al., 2013; Magrané et al., 2014). This process is related to a clear alteration in mitochondrial morphology, with mitochondria of transgenic mice appearing shorter in length and fragmented. Further, it seems to be specific for motor neurons, as it is recognizable in the ventral roots motor axons, but not in sensory neurons of the dorsal roots (Magrané et al., 2014). In the G93A-SOD1 mouse, in particular, this event is already evident at 15 days of age, suggesting that an early fragmentation of mitochondria might underlie retrograde transport defects. Interestingly, early, pre-symptomatic defects in mitochondrial transport and morphology were also observed in the skeletal muscle of G93A-SOD1 overexpressing mice. Interestingly, inhibition of mitochondrial fission by a Drp-1 inhibitor restored both mitochondrial network and transport (Luo et al., 2013), thus extending to muscle cells, that might well play an important role in ALS pathogenesis, the relevance of mitochondrial dynamics defects. In an A315T-TDP43 transgenic mouse, however, mitochondrial fragmentations seems to occur significantly later than mitochondrial transport defects, suggesting the existence of alternative mechanisms accounting for mitochondrial dysfunction in the two mouse models (Magrané et al., 2014). Yet, when cultured motor neurons were transfected with both wild-type and the M333V ALS-linked mutant of TDP-43 in the presence of an overexpressed Mfn2, a rescue in both length and density of mitochondria was observed and their transport along axons and dendrites recovered (Wang et al., 2013). On these grounds, it is therefore clear that mitochondria alterations, including defective mitochondrial dynamics, are tightly linked to ALS pathogenesis.

## Mitochondria involvement in ALS: genetic evidence open a new scenario

Nevertheless, whether mitochondria have a causative, primary role in the disease has always been debated. Recent experimental evidence, however, has provided significant genetic basis to support the conclusion that mitochondria degeneration is deeply involved in the pathogenic mechanisms, at least in some forms of the disease, and more generally, that motor neurons are particularly vulnerable to defective mitochondrial function.

In a screening of muscle biopsies from patients belonging to a family that presented late-onset myopathy, coupled to motor and cognitive phenotypes, including motor neuron disease and FTD-like symptoms, Bannwarth et al. have identified a missense mutation (S59L) in the nuclear *CHCHD10* gene, which encodes for a mitochondrial-resident protein (Bannwarth et al., 2014). An identical missense mutation was pinpointed by the same authors in a screening of 21 families characterized by ALS/FTD phenotypes, leading to the hypothesis that variations in the *CHCHD10* gene is a cause of ALS and FTD. These findings promptly led the same group, as well as other labs, to screen for variants in *CHCHD10* gene in the ALS/FTD population. Results from these screenings confirmed the existence of this variant in different ALS pedigrees (Chaussenot et al., 2014; Johnson et al., 2014; Müller et al., 2014). Further, three novel mutations (R15L, P34S and G66V) were identified in patients with ALS/FTD or ALS only, which strengthened the connection between *CHCHD10* mutations and these diseases (Chaussenot et al., 2014; Johnson et al., 2014; Müller et al., 2014). Interestingly, a double missense mutation (R15S/G85R) in the coding region of *CHCHD10* has been identified in patients suffering from autosomal dominant mitochondrial myopathy, a disease characterized by morphological and biochemical alterations of mitochondria (Ajroud-Driss et al., 2015). Thus, mitochondrial function could be profoundly affected by mutations in this gene, independently from the tissue affected.

Although further analysis in larger populations is needed to confirm the frequency of these mutations in the familial/sporadic ALS population, and to make the genetic link between *CHCHD10* and ALS more robust (van Rheenen et al., 2014), these findings might represent a landmark discovery in ALS research, because for the first time a gene coding for a mitochondrial protein has been identified as a possible cause for the disease, which eventually implies mitochondrial dysfunction as a primary trigger of ALS. Furthermore, they also provide additional circumstantial evidence that alteration in mitochondrial dynamism is involved in ALS pathogenesis. Indeed, although the function of *CHCHD10* protein is still almost uncharacterized, preliminary experiments and structural analogies with proteins of the CHCH family suggest that it might be involved in the regulation of mitochondrial function in general, and in mitochondria morphology processes in particular.

*CHCHD10* shows a prominent mitochondrial localization (Bannwarth et al., 2014; Ajroud-Driss et al., 2015), and sub-mitochondrial fractionation coupled to protease digestion showed that *CHCHD10* is located in the intermembrane space of mitochondria (Bannwarth et al., 2014). Overexpression of the mutant S59L-*CHCHD10* in HeLa cells profoundly affected mitochondrial structure, inducing a clear fragmented morphology with disorganization of cristae structure and matrix condensation (Bannwarth et al., 2014). This observation is in line with the effect that the G58R mutant found in autosomal dominant mitochondrial myopathy patients produced in cultured human cells (Ajroud-Driss et al., 2015), suggesting that the mitochondria-induced morphological defects that distinguish *CHCHD10* mutations are a common trait of highly metabolic tissues such as muscle and the nervous system.

**Figure 1:**
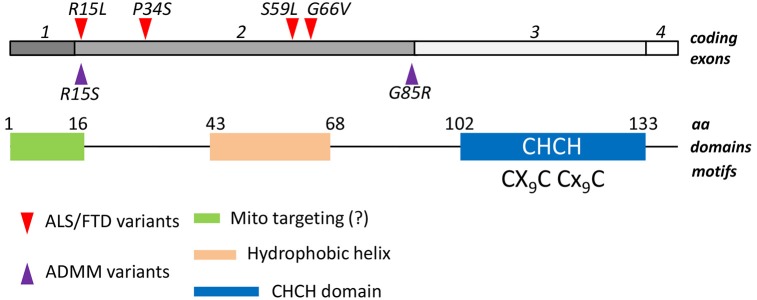
***Structural features of CHCHD10***. Four exons of the human *CHCHD10* gene encode for a 142 aa mature protein. A coiled helix coiled helix domain (CHCH) is present in the C-terminal half of the polypeptide. A twin CX9C domain, which is targeted by the Mia40/Erv1 mechanism of oxidative import of mitochondrial proteins, is also present. A putative mitochondrial targeting sequence in the N-terminus, and a central hydrophobic helix have been also described. Mutations associated to ALS/FTD phenotypes (red triangles) and to Autosomal Dominant Mitochondrial Miopathy (ADMM, purple triangles) are reported. They all fall in the second exon.

As the others members of the CHCH protein family, *CHCHD10* contains a coiled coil-helix-coiled coil-helix (CHCH) fold that is stabilized by disulfide bonds formed by twin CX(9)C motifs (Figure 1). The CX_n_C motif has emerged as a central attribute underlying the import in mitochondria of proteins lacking canonical organelle targeting signals. The mechanism has been characterized in detail: cysteine-containing motifs are targeted by the mitochondrial intermembrane space assembly (MIA) machinery, that oxidizes cysteine residues, and drive the electrons from reduced and unfolded proteins to the respiratory chain. As a result, proteins reach their folded, oxidized state and are entrapped inside the intermembrane space. The reaction is carried on through the intervention of an oxidoreductase, MIA40, and a sulphhydryl oxidase, Erv1 (Mesecke et al., 2005). Since the redox milieu of mitochondria is clearly altered in ALS conditions (Ferri et al., 2006), it is tempting to speculate that either mutations in the gene, or alteration in the mitochondrial redox balance might affect the proper mitochondrial import of *CHCHD10*, eventually leading to mitochondria dysfunction. However, the mutations in *CHCHD10* that have been described so far do not seem to alter the mitochondrial localization of the protein (Bannwarth et al., 2014; Ajroud-Driss et al., 2015), which is in agreement to the fact that these mutations do not fall into the CHCH domain nor in a putative N-terminal mitochondrial localization signal, that is predicted by bioinformatical analysis (Figure 1).

Although a more detailed analysis of mitochondrial localization of the mutant protein is needed, it is reasonable to imagine that the impaired mitochondrial function by mutant *CHCHD10* is consequent to an altered protein function, which is to date still unknown. Circumstantial evidence suggest that this function might be closely related to the control of mitochondrial morphology and the fusion/fission process. Indeed, mutant *CHCHD10* overexpression in HeLa cells altered mitochondrial shape, inducing a fragmented phenotype, as already mentioned. Moreover, immunoelectron microscopy showed that *CHCHD10* is located in close proximity of mitochondrial cristae junctions, and overexpression of the S59L mutant induced cristae loss, disorganization and dilation (Bannwarth et al., 2014). Further, *CHCHD3*, a member belonging to the same CHCH mitochondrial family of proteins, has a possible function in the regulation of cristae morphology as well (Darshi et al., 2011), and cristae shape is tightly linked to the assembly of respiratory chain complexes into quaternary supercomplexes that represent the functional mitochondrial respiratory units (Acín-Pérez et al., 2008; Cogliati et al., 2013). Accordingly, *CHCHD3* interacts with OPA1, and down-regulation of *CHCHD3* in HeLa cells resulted in fragmentation of the mitochondrial network and a significant remodeling of the cristae architecture, two functions that OPA1 controls through independent mechanisms (Frezza et al., 2006). Interestingly, *CHCHD3* is significantly decreased in NSC34 motor neuron-like cells overexpressing a mutant SOD1 that is associated to familial ALS, further suggesting a possible wider role of CHCH-containing family of mitochondrial proteins in the pathogenesis of the disease (Fukada et al., 2004).

In conclusion, a renewed interest to altered mitochondria physiology in ALS comes from genetic evidence of a link between an ALS/FTD phenotype in patients and mutations in *CHCHD10* gene. Further studies are needed to assess whether an altered mitochondrial dynamism is a cause or a consequence of mitochondrial functional derangement, whether it is common to all forms of ALS and whether interventions aimed to restore a correct dynamism may represent a therapeutic option.

## Conflict of interest statement

The Review Editor Silvia Campello declares that, despite being affiliated to the same institution as authors Alessia Mirra and Maria Teresa Carrì, the review process was handled objectively and no conflict of interest exists. The authors declare that the research was conducted in the absence of any commercial or financial relationships that could be construed as a potential conflict of interest.
